# Coronary CT Angiography in Coronary Artery Disease: Correlation between Virtual Intravascular Endoscopic Appearances and Left Bifurcation Angulation and Coronary Plaques

**DOI:** 10.1155/2013/732059

**Published:** 2013-12-28

**Authors:** Zhonghua Sun

**Affiliations:** Discipline of Medical Imaging, Department of Imaging and Applied Physics, Curtin University, GPO Box U1987, Perth, WA 6845, Australia

## Abstract

The aim of this study is to investigate the relationship between intraluminal appearances of coronary plaques and left coronary bifurcation angle and plaque components using coronary CT virtual intravascular endoscopy (VIE). Fifty patients suspected of coronary artery disease undergoing coronary CT angiography were included in the study. The left bifurcation angle in patients with diseased left coronary artery which was measured as 94.3° ± 16.5 is significantly larger than that in patients with normal left coronary artery, which was measured as 76.5° ± 15.9 (*P* < 0.001). Irregular VIE appearances were found in 10 out of 11 patients with mixed plaques in the left anterior descending (LAD) and left circumflex (LCx), while, in 29 patients with calcified plaques in the LAD and LCx, irregular VIE appearances were only noticed in 5 patients. Using 80° as a cut-off value to determine coronary artery disease, smooth VIE appearances were found in 95% of patients (18/19) with left bifurcation angle of less than 80°, while irregular VIE appearances were observed in nearly 50% of patients (15/31) with left bifurcation angle of more than 80°. This preliminary study shows that VIE appearances of the coronary lumen are directly related to the types of plaques.

## 1. Introduction

During the past decade, coronary CT angiography has been increasingly used as an effective less invasive modality for diagnosis of coronary artery disease (CAD) due to its improved spatial and temporal resolution [1–3]. In addition, to accurately assess the degree of coronary stenosis, coronary CT angiography enables visualization of atherosclerotic plaques [4–6]. This is represented in its ability to identify the location and distribution of plaques in the coronary arteries and characterize the type of plaques as well as assess the plaque composition [4–6]. Studies have demonstrated the feasibility of differentiation between calcified, noncalcified, or mixed plaques based on differences in CT attenuation [4, 7]. Since the noninvasive coronary CT angiography is continuously expanding, the technique will increasingly be used to identify patients at either low or high risk of developing cardiac events, based on the composition of plaques and coronary lumen changes [7, 8].

Therefore, the potential value of coronary CT angiography to differentiate between various patterns of atherosclerotic lesions needs to be further explored, as it may prove to provide additional information for risk stratification and patient management of CAD. Traditional assessment of coronary plaques is based on 2D axial and multiplanar reformatted images; however, the main limitation of this visualization is the lack of direct intraluminal views of plaque appearances and corresponding coronary wall changes. This limitation is complemented by a 3D reconstruction tool, virtual intravascular endoscopy (VIE) which shows diagnostic applications in cardiovascular disease [9–14].

Since the angulation of the bifurcation of left coronary artery might have an effect on hemodynamic shear stress and consequently on plaque size and development, the bifurcation angles in relation to the plaques and development of CAD have been studied. It is proposed and reported in previous studies that a wider bifurcation angle might be related to higher turbulence and low shear stress, whereas a narrow angle might be more prone to present laminar flow [4, 15]. This study has twofold purposes: to demonstrate the diagnostic applications of 3D VIE in the visualization and characterization of intraluminal appearances of coronary plaques in patients with different plaque components and variable left coronary bifurcation angles and to determine if there is a direct correlation between 3D VIE appearances of coronary wall and left bifurcation angle and types of plaques. It is expected that research findings of this study will contribute to accurate assessment of plaque morphology with corresponding coronary wall changes, risk stratification and patient management.

## 2. Materials and Methods

This study is a retrospective analysis of images in patients who were referred for coronary CT angiography by their physicians as a routine procedure for clinical diagnosis; thus, no institutional review board (IRB) was required. Patient's details were deidentified in all of the images. Patient informed consent was waived.

### 2.1. Patient Data and Coronary CT Angiography

Fifty patients (31 men, 19 women, mean age: 55.6 years ±8.9) suspected of CAD undergoing coronary CT angiography examinations were included in the study. Coronary CT angiography was performed with a 64-slice scanner (GE Medical Systems, Lightspeed VCT, GE Healthcare, Milwaukee, WI, USA) in 35 patients, 256-slice scanner (Brilliance iCT, Philips Healthcare, Cleveland, OH, USA) in 10 patients, and 320-slice CT (Toshiba Aquilion One Dynamic Volume CT, Tochigi-ken, Japan) in the remaining 5 patients. The scanning protocols for 64-, 256-, and 320-slice CT coronary angiography were as follows: beam collimation 64 × 0.625 mm, pitch 0.2–0.26, reconstruction interval of 0.4 mm, with tube voltage of 120 kVp and tube current modulation ranging from 300 to 650 mAs; 256 × 0.625 mm, pitch 0.2, reconstruction of 0.4 mm, with tube voltage of 120 kVp and tube current modulation of 500 mAs; 320 × 0.5 mm, pitch 1.0, reconstruction interval of 0.4 mm, tube voltage of 120 kVp with tube current modulation of 320 mAs. Patients with renal insufficiency presenting with elevated serum creatinine levels (>1.5 mg/dL) and documented hypersensitivity to iodinated contrast materials and treated with coronary artery bypass grafts or coronary stents were excluded from the study.

Nonionic contrast medium (Iopamiro 370 or Visipaque 320, 60–80 mL) was injected onto the antecubital vein at 3–5 mL/s for the first 40–60 mL and 3–3.5 mL/s for the remaining 20 mL followed by 50 mL of saline chasing at 4-5 mL/s, and the scan was performed with a bolus tracking technique with CT attenuation of 200–220 HU as the triggering threshold at the ascending aorta to initiate the scan.

Axial images were reconstructed with a slice thickness of 0.5–0.625 mm in 0.4 mm increment resulting in isotropic volume data with a voxel size ranging from 0.4 × 0.4 × 0.4 to 0.6 × 0.6 × 0.6 mm^3^. Retrospective electrocardiographic-gated protocol was used to acquire the volume data achieving a temporal resolution of 135–165 ms in the centre of the gantry rotation. Volume data were reconstructed at 70–80% R-R interval to minimize the artefacts. For patients with a heart rate more than 70 beats per minute (bpm), a beta-blocker was used to slow down the heart rate prior to the CT scan.

### 2.2. Measurements of Left Bifurcation Angle and Coronary Artery Diameters

**Figure 1:**
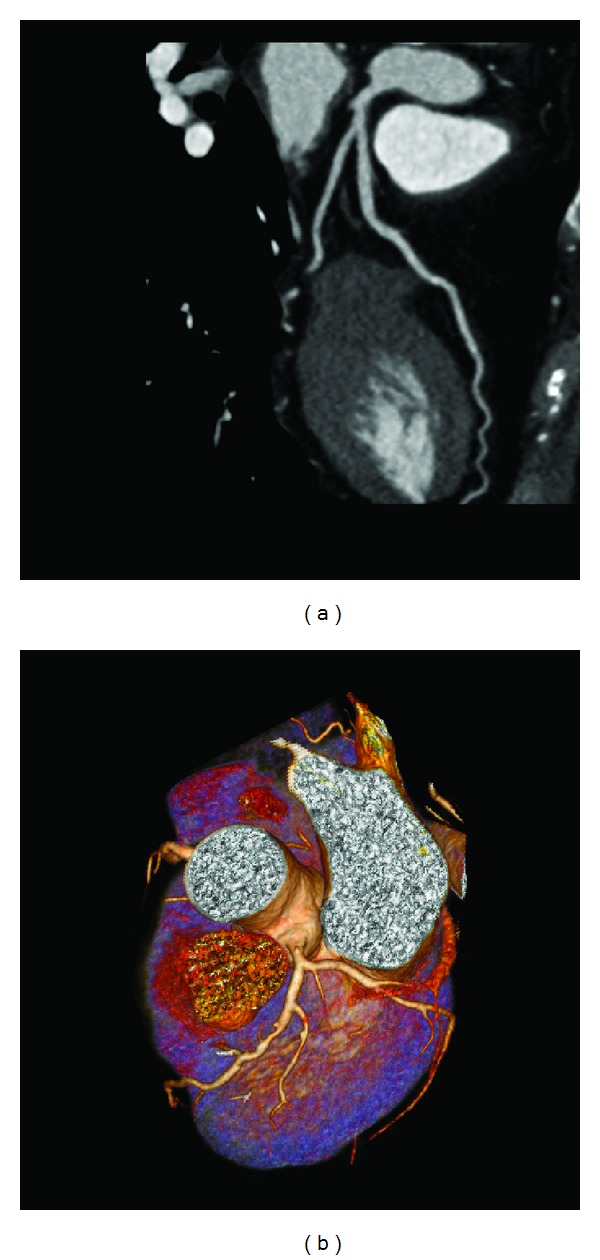
Curved planar reformatted image (a) shows that the left bifurcation angle is measured as 40.3° in a 54-year-old woman with a normal left coronary artery. 3D volume rendering (b) shows that the left bifurcation angle is measured as 104.5° in 71-year-old man with left coronary disease.

3D volume rendering and curved planar reformatted images were generated for the measurement of each bifurcation angle between the left anterior descending (LAD) and left circumflex (LCx) (Figure 1).

Diameters of LAD and LCx were measured and compared on curved planar reformatted images in each patient. Measurements were performed to obtain the maximal diameters of coronary lumens at either normal or atherosclerotic regions with aim of indicating the correlation between coronary dimensional changes and presence of plaques; thus, measurements were avoided at the stenotic regions. Maximal diameters of LAD and LCx were repeatedly measured 3 times at each anatomic location, and the average values were used to reduce intraobserver disagreement.

### 2.3. Generation of Virtual Intravascular Endoscopy Images

**Figure 2:**
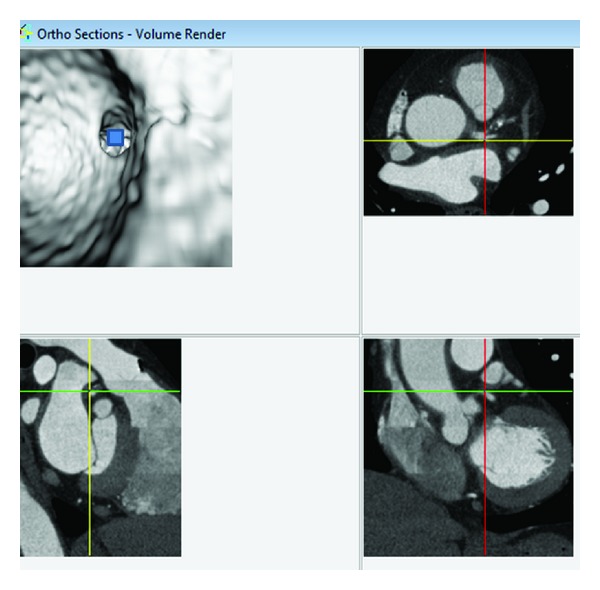
Visualization of anatomic structures on virtual intravascular endoscopy (VIE) image. A VIE image is generated to look at the ostium of left coronary artery (top left), and corresponding orthogonal views (axial on top right, coronal and sagittal views on the bottom right and left, resp.) confirm the exact location of the structure. The blue box indicates the viewing position placed on the VIE image.

Original DICOM data (digital imaging and communication in medicine) were transferred to a workstation equipped with Analyze V 11.0 (AnalyzeDirect, Inc., Lenexa, KS, USA) for generation of 3D VIE images. Postprocessing of CT data was performed with a CT number thresholding technique, which was described before [9–11]. In summary, the first step was to measure the CT attenuation at the main coronary arteries, namely, right and left coronary arteries, to determine the threshold that was used to remove the contrast-enhanced blood from the coronary artery. Then, the CT threshold value which was measured between 150 HU and 300 HU in the first step was applied to generate the intraluminal views of coronary artery ostium, lumen surface, and coronary plaques. Since coronary artery is relatively small in diameter (3–5 mm in the proximal segment), intraluminal appearance of the coronary ostium and plaques visualized on VIE images need to be correlated with corresponding orthogonal views to determine the exact anatomic details (Figure 2). This ensures accurate identification of the location of both normal anatomic structures and pathological changes.

### 2.4. VIE Characterization of Coronary Plaques

**Figure 3:**
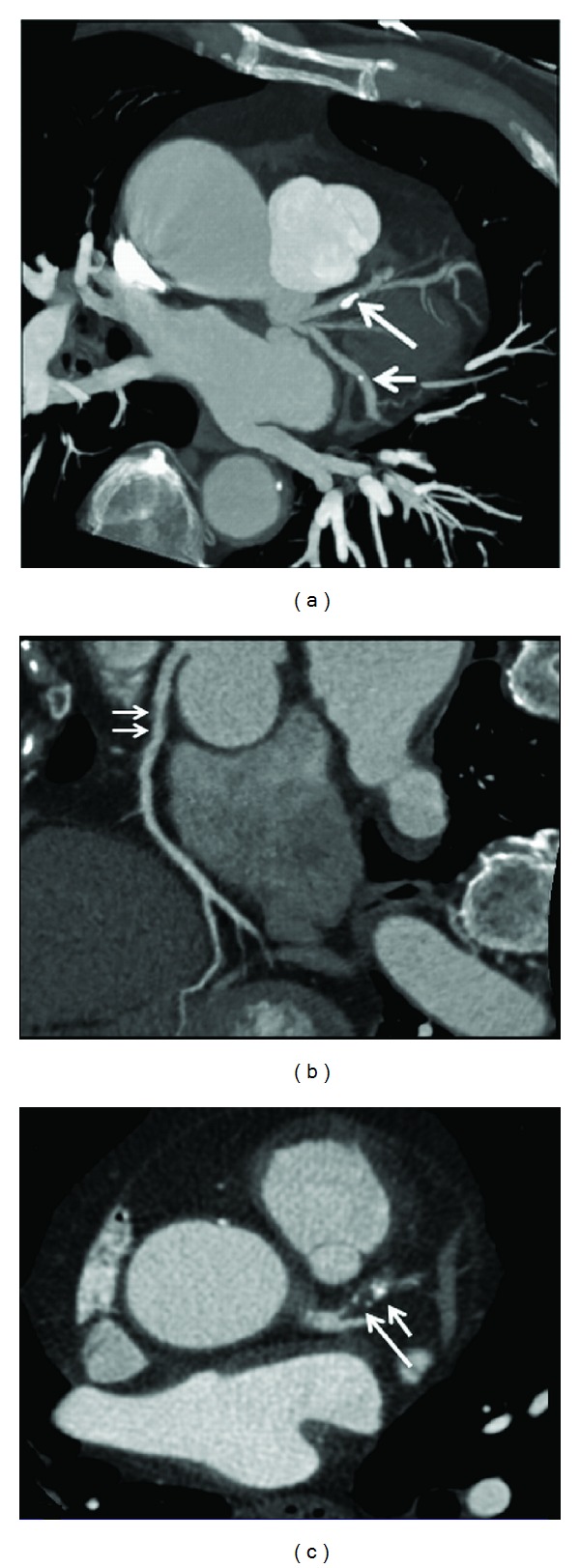
Maximum-intensity projection shows that calcified plaques are present at the proximal segment of left anterior descending (long arrow) and midsegment of left circumflex (short arrow) (a). Curved planar reformation shows a noncalcified plaque at the proximal segment of right coronary artery ((b) arrows). 2D axial image demonstrates a mixed plaque at the proximal segment of left anterior descending (c), with short arrow referring to the calcified component and long arrow to the noncalcified component within the plaque.

Coronary artery plaque was classified into the following three types based on the CT attenuation [16]: calcified plaques indicate plaques with high density (Figure 3(a)); noncalcified plaques refer to plaques having lower density compared with the contrast-enhanced vessel lumen (Figure 3(b)); mixed plaques indicate plaques with noncalcified and calcified elements within a single plaque or within a segment of the coronary artery (Figure 3(c)).

### 2.5. Statistical Analysis

All data were entered into SPSS V 19.0 (SPSS Inc., Chicago, IL, USA) for statistical analysis. Continuous variables were expressed as mean value ± SD. Comparisons were performed using one sample *t*-test. A *P* value less than 0.05 was defined as a statistically significant difference.

## 3. Results

VIE was successfully generated in all of these patients with clear demonstration of the intraluminal appearances of coronary lumen and plaques in patients with both normal coronary artery and diseased coronary artery.

### 3.1. Plaque Distribution and Left Bifurcation Angle

Since the left main stem was free of involvement of coronary plaques in more than 90% of the patients, lesions in the left coronary artery branches were classified based on distribution in the LAD and LCx branches. Coronary plaques were found to be present in 33 patients with atherosclerotic lesions located at the LAD in 19 patients and at both LAD and LCx in 14 patients (Table 1). Most of the plaques belonged to calcified or mixed type of plaques, while noncalcified plaques were only observed in one case, as shown in the table.

**Figure 4:**
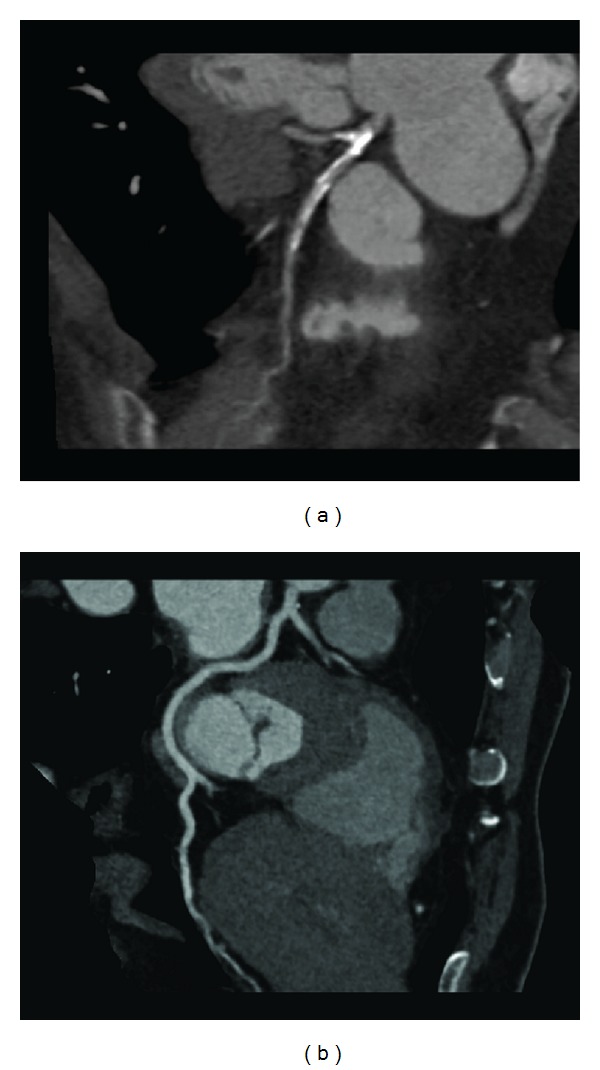
Curved planar reformatted images show that the left bifurcation angle is measured as 88.5° in a 58-year-old woman with extensive calcified plaques in the left anterior descending and left circumflex (a), while, in another 46-year-old man, the angle is measured as 71.3° with normal left coronary artery (b).

The mean bifurcation angle between LAD and LCx was measured as 88.1° ± 18.3 (range, 40.3°, 134.5°). The bifurcation angle in patients with diseased left coronary artery which was measured as 94.3° ± 16.5 is significantly wider than that in patients with normal left coronary artery, which was measured as 76.5° ± 15.9 (*P* < 0.001) (Figure 4). Of 33 patients with left coronary disease, 25 (76%) had a bifurcation angle >80° and 91% of these patients with diseased LAD/LCx had a bifurcation angle >80°. This indicates a strong correlation between wider angulation and formation of coronary plaques.

### 3.2. Left Coronary Diameters in relation to the Bifurcation Angle

**Figure 5:**
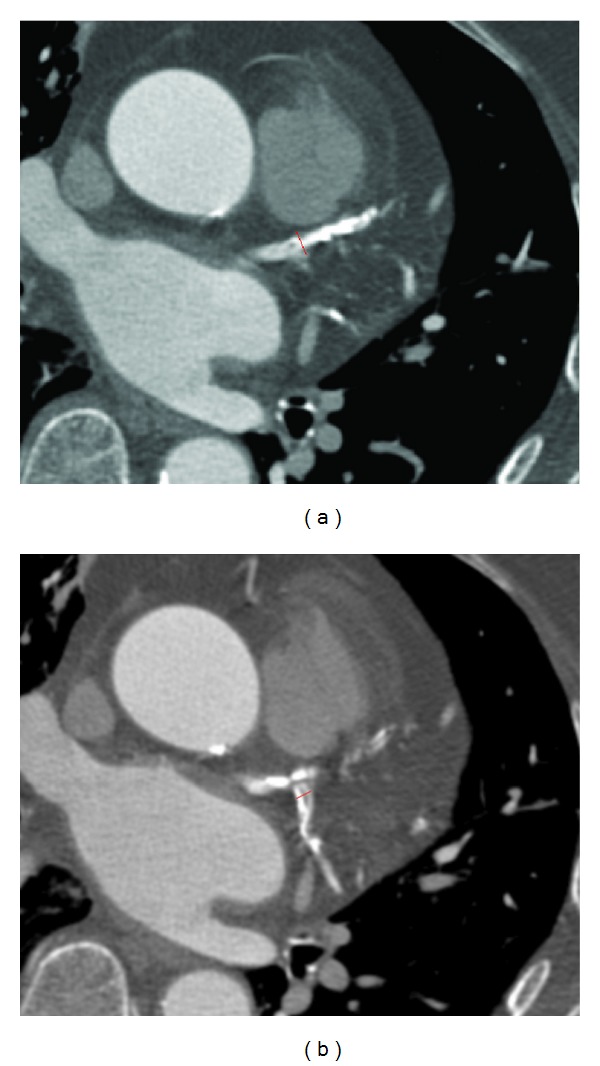
Measurement of the left coronary artery diameter is shown on 2D axial image in a 52-year-old man with significant coronary stenosis. The left anterior descending (a) and left circumflex branches (b) are measured as 6.1 mm and 3.7 mm in the proximal segments due to presence of extensively calcified plaques at both artery branches.

The mean diameters of LAD and LCx among all the patients were measured as 3.9 ± 0.8 mm (range, 2.2 mm, 6.1 mm) and 2.9 ± 0.6 mm (range, 1.6 mm, 4.2 mm), respectively. The mean diameters of LAD and LCx measured in patients with the normal left coronary artery were 3.2 ± 0.5 (range, 2.2 mm, 4.2 mm) and 2.4 ± 0.4 mm (range, 1.9 mm, 3.1 mm), and this is significantly smaller than those measured in patients with the diseased LAD and LCx (*P* < 0.001) (Figure 5), which were 4.1 ± 0.8 mm (range, 2.8 mm, 6.1 mm) and 3.1 ± 0.6 mm (range, 1.6 mm, 4.2 mm), respectively.

A bifurcation angle of 80° was used as a cut-off value to determine the presence and extent of left coronary artery disease. The mean diameters of LAD and LCx in patients with diseased left coronary with >80° bifurcation angle were measured as 4.1 ± 0.9 mm (range, 2.2 mm, 6.1 mm) and 3.1 ± 0.6 mm (range, 1.5 mm, 4.2 mm). In contrast, the mean diameters of LAD and LCx in patients with diseased left coronary disease with <80° bifurcation angle were measured as 3.4 ± 0.5 mm (range, 2.5 mm, 4.5 mm) and 2.6 ± 0.5 mm (range, 1.6 mm, 3.4 mm), respectively, reaching statistically significant difference (*P* = 0.03).

### 3.3. VIE Appearances of Coronary Lumen

The appearances of coronary lumen visualized on VIE images were classified into smooth and irregular appearances, depending on the type and composition and extent of the plaques. Moreover, the position or location of the plaques in relation to the artery wall was assessed in terms of eccentric or concentric appearance or luminal changes regarding the involvement of superior or inferior portion of the wall. This information reflects the remodelling of the coronary wall due to deposition of plaques, which in turn will contribute to the subsequent cardiac events if the plaques are found to be unstable or vulnerable.

**Figure 6:**
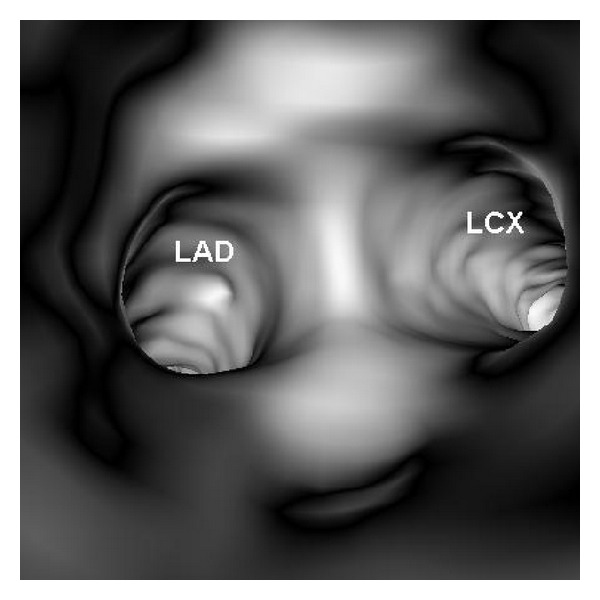
VIE visualization of normal coronary artery wall. Smooth intraluminal appearance is observed with clear demonstration of the coronary ostia of left anterior descending (LAD) and left circumflex (LCx).

**Figure 7:**
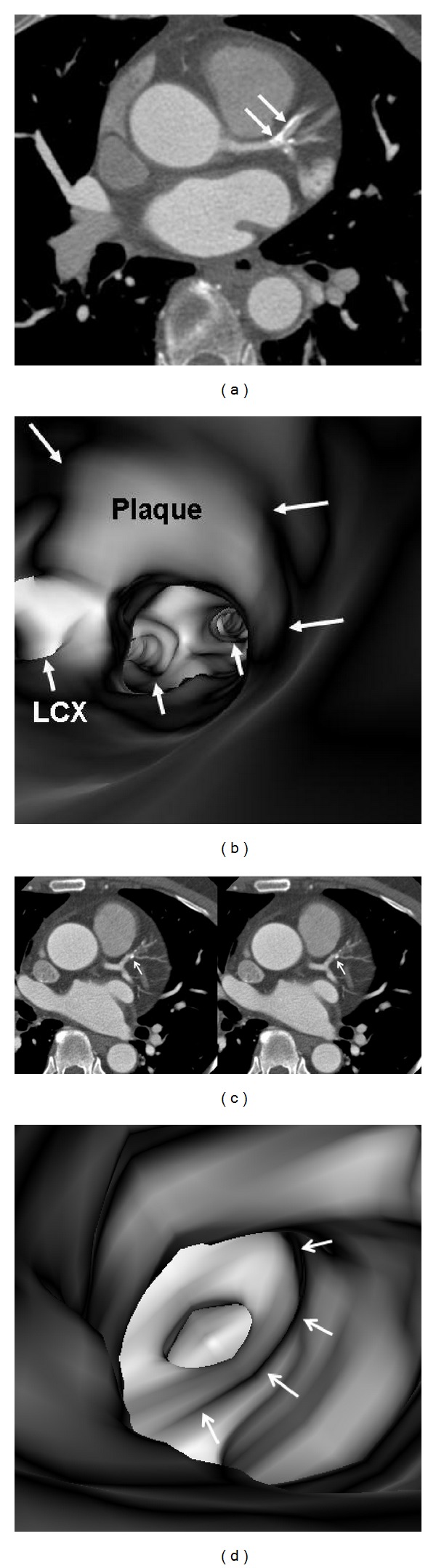
VIE appearance of calcified plaques. An eccentric calcified plaque is formed in the left anterior descending on a 2D axial image ((a) arrows), and corresponding VIE shows the smooth protruding appearance in the superior lumen of left anterior descending ((b) long arrows). The plaque is close to the ostium of left circumflex (LCx) and covers the other ostia of left coronary side branches (short arrows). A concentric plaque is present in the left anterior descending in another patient on 2D axial images ((c) arrows), and corresponding VIE shows intraluminal smooth appearance around the coronary lumen ((d) arrows).

**Figure 8:**
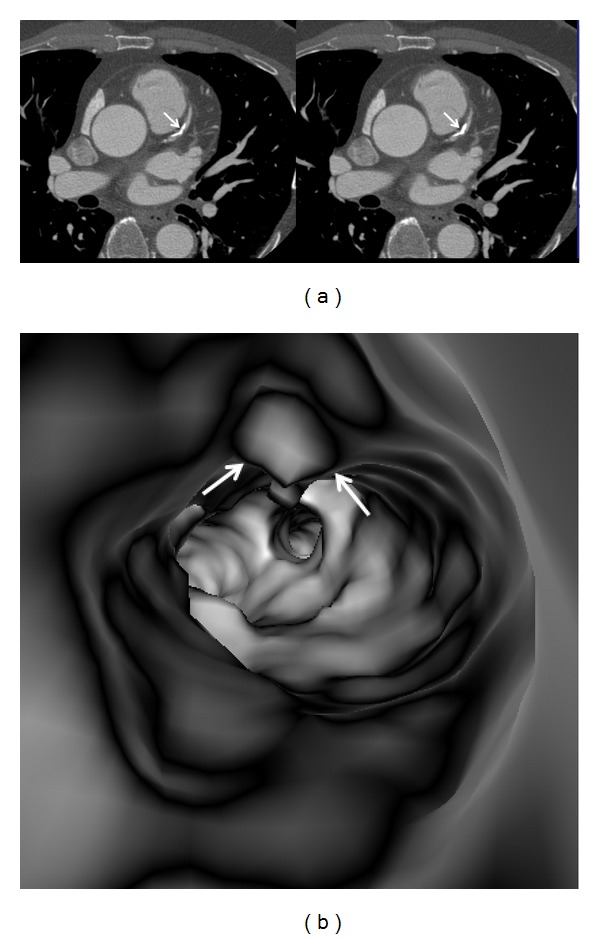
A heavily calcified plaque is present at the proximal segment of left anterior descending ((a) arrows). VIE shows irregular intraluminal appearance ((b) arrows).

**Figure 9:**
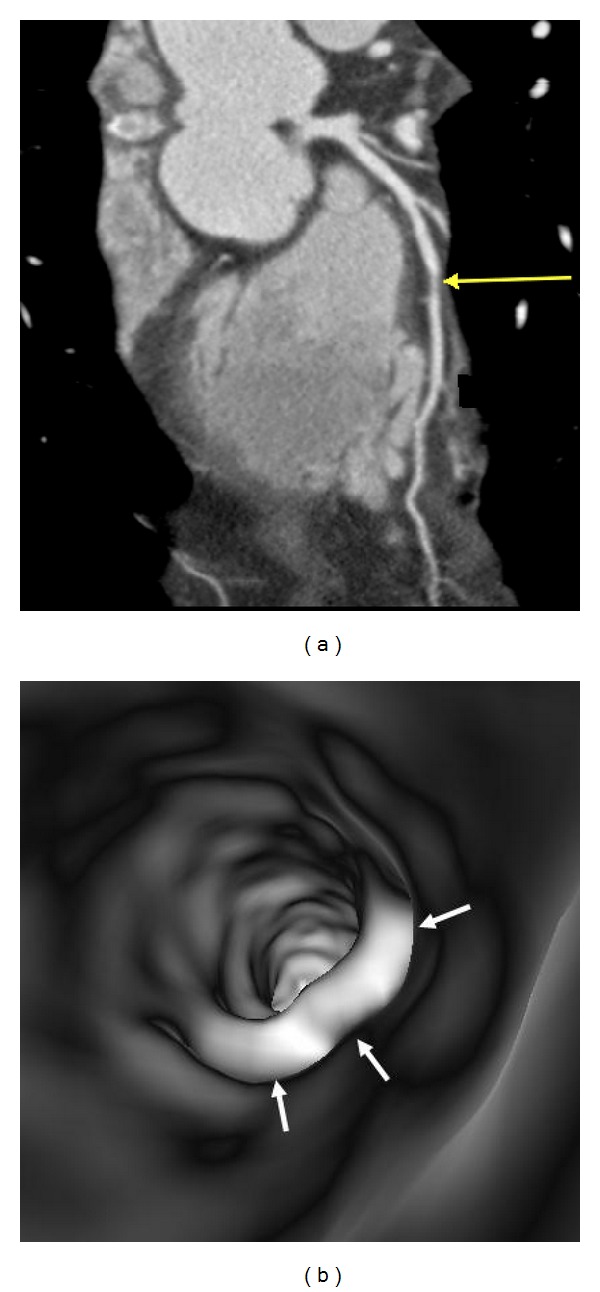
VIE appearance of noncalcified plaques. A noncalcified plaque is found in the midsegment of left anterior descending on a curved planar reformatted image ((a) arrow), and corresponding VIE confirms the smooth protruding appearance arising from the inferior lumen of the coronary artery wall ((b) arrows).

In the absence of coronary plaques or atherosclerotic changes, the coronary wall appears smooth with clear demonstration of the ostial configuration and lumen on VIE visualization (Figure 6). When the plaques are present in the artery wall, different configurations are noticed, depending on the composition of the plaques. For calcified plaques, VIE shows smooth protruding appearance in the coronary wall either surrounding the ostium eccentrically or around the coronary wall concentrically (Figure 7) or irregular appearance due to heavily calcified plaques (Figure 8). For noncalcified plaques, a protruding sign arising from the artery wall can be visualized on VIE (Figure 9).

### 3.4. Correlation of VIE Appearances with Different Types of Plaques

**Figure 10:**
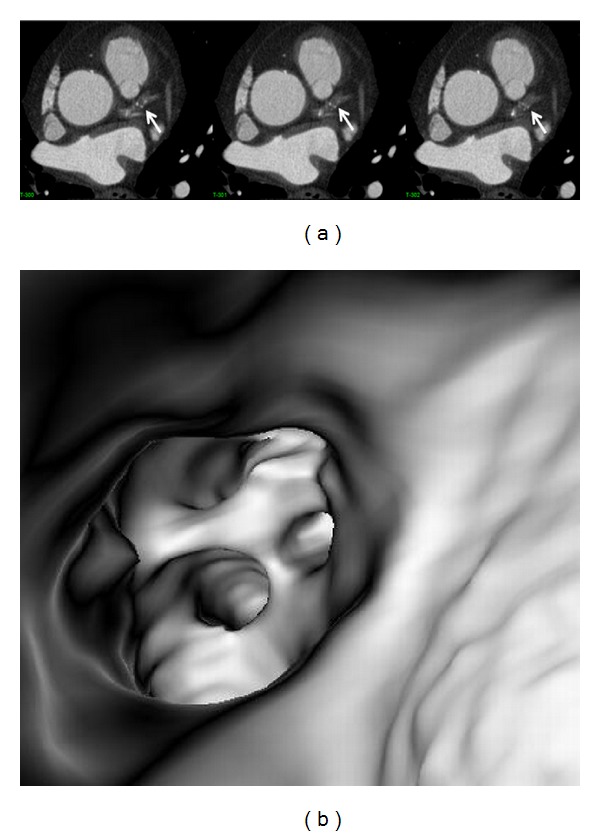
A series of 2D axial images show mixed plaques with the majority of the components representing noncalcified plaque at the proximal segment of left anterior descending ((a) arrows). Corresponding VIE shows irregular lumen change due to different plaque components (b).

**Figure 11:**
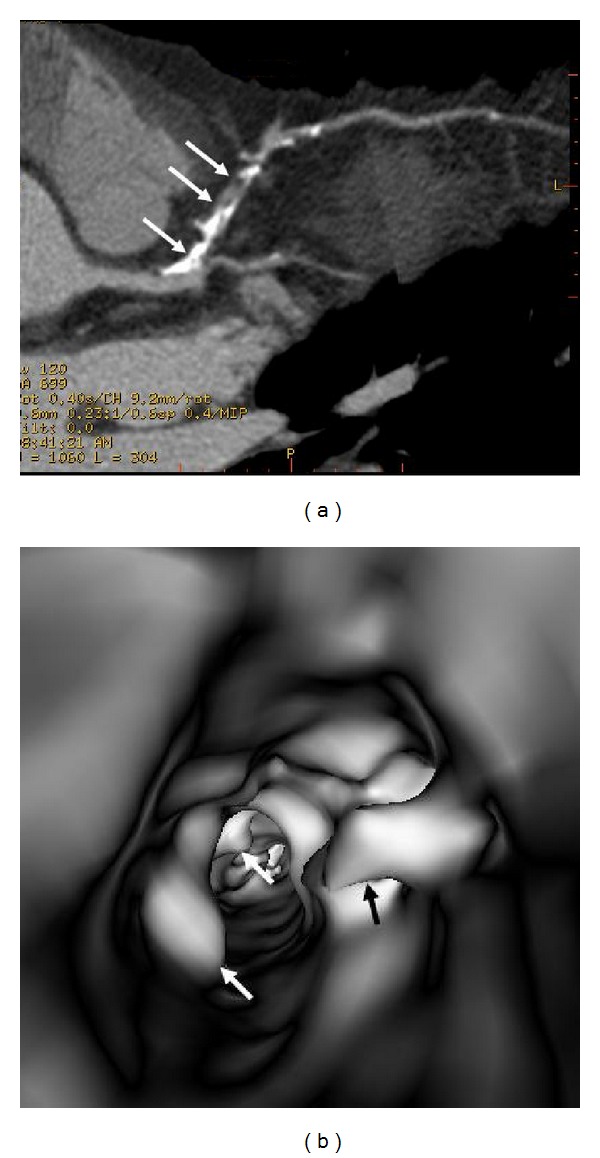
VIE appearance of mixed plaques within a coronary segment. Extensive calcification and noncalcified component are found in the left anterior descending representing the mixed plaques on a curved planar reformatted image ((a) arrows). VIE shows irregular intraluminal changes due to different compositions within the plaques ((b) arrows).

**Figure 12:**
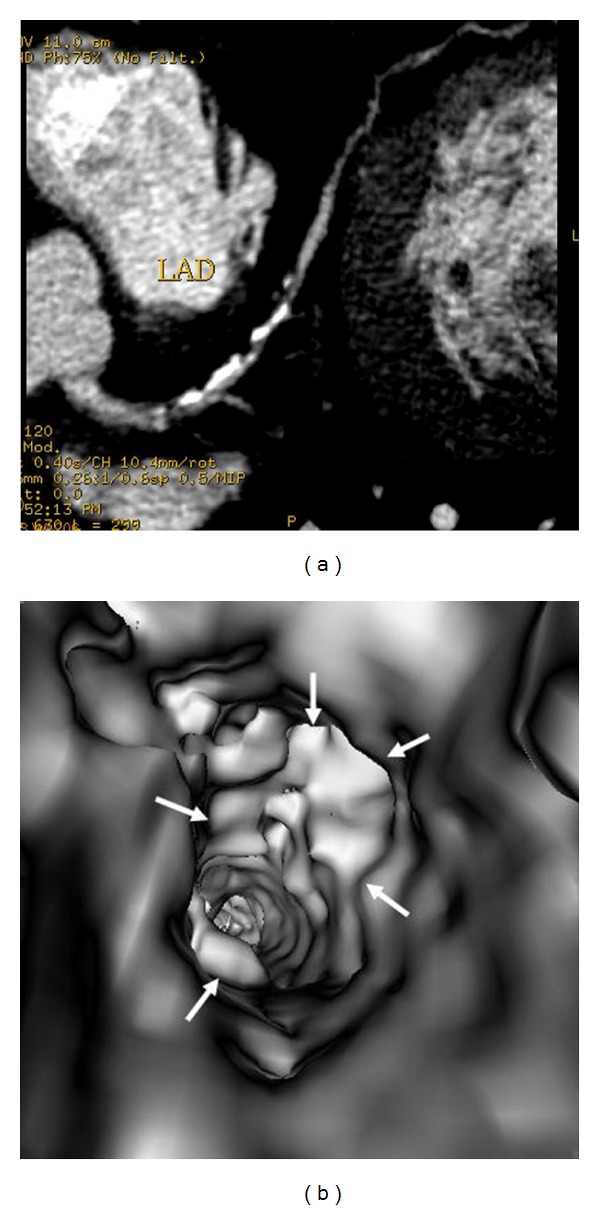
VIE appearance of extensively calcified plaques. More than 50% lumen stenosis is noticed in the left anterior descending on a curved planar reformatted image (a). VIE reveals the significant stenosis with plaque surrounding the coronary wall of left anterior descending ostium with irregular intraluminal appearances ((b) arrows).

**Figure 13:**
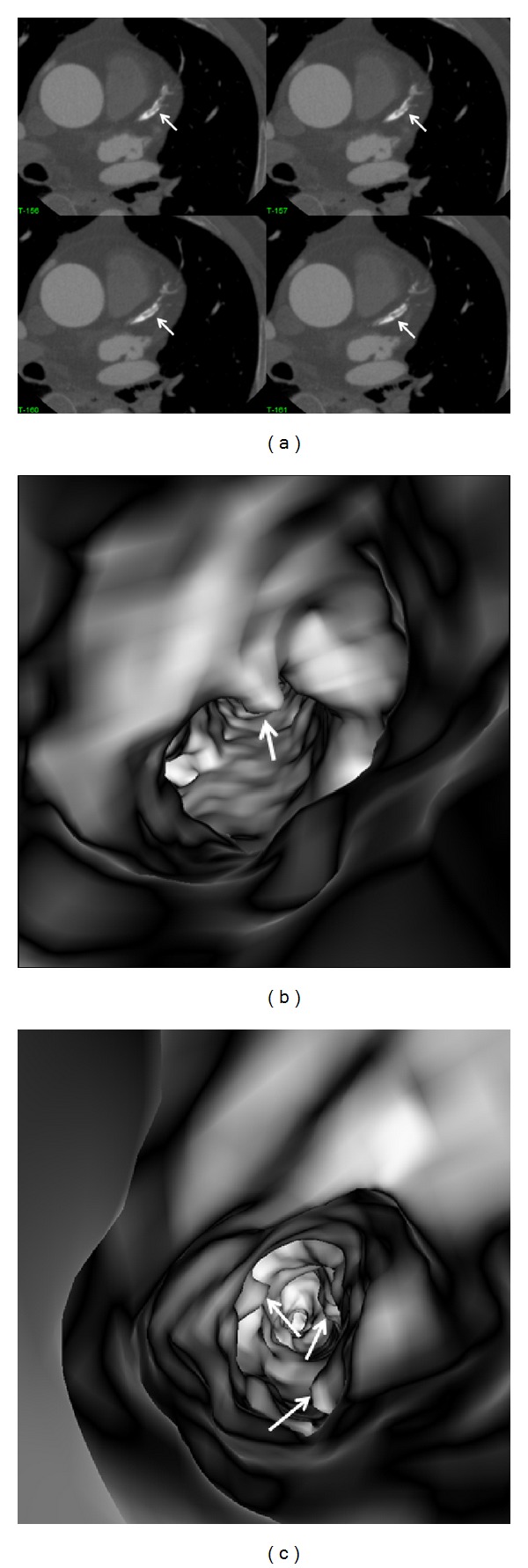
2D axial images show the extensively calcified plaques in the left anterior descending ((a) arrows). VIE demonstrates irregular intraluminal changes at the proximal segment ((b) arrow) and midsegment of left anterior descending ((c) arrows) with significant lumen stenosis.

**Figure 14:**
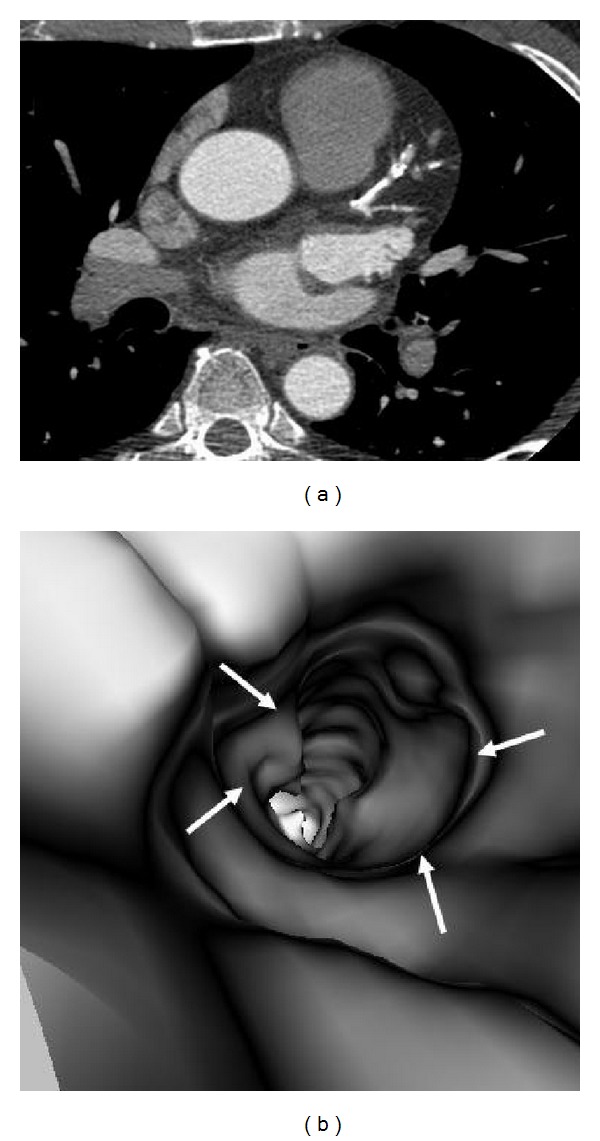
Extensively calcified plaques on a 2D axial image show more than 90% stenosis (due to blooming artefacts) in the left anterior descending (a). VIE confirms that only 60% lumen stenosis with smooth intraluminal appearance ((b) arrows).

Table 1 shows VIE appearances in relation to the left bifurcation angle and different types of plaques. Irregular VIE appearances of the coronary wall were most commonly observed in the mixed type of plaques as shown in the table. Of 11 patients with mixed plaques in the LAD and LCx, irregular VIE appearances were found in 10 patents (91%) (Figures 10 and 11). In contrast, of 21 patients with only calcified plaques in the LAD and LCx, irregular VIE appearances were only seen in 5 patients (24%), while, in the remaining patients, VIE visualization showed smooth intraluminal coronary wall. The irregular VIE appearances due to calcified plaques were commonly seen in the coronary arteries with extensively calcified plaques due to the remodelling of the artery wall caused by plaques formed along the coronary artery (Figures 12 and 13), although regular and smooth appearances were also seen in some of the extensively calcified plaques (Figure 14).

### 3.5. Correlation of VIE Appearances with Left Bifurcation Angle

Similarly, a bifurcation angle of 80° was used as a cut-off value to demonstrate the relationship between VIE appearances and bifurcation angle. As shown in the table, 31 patients had a bifurcation angle of more than 80°, with irregular VIE appearances being observed in nearly 50% of these patients (15/31) (Figures 10, 11, and 12). In contrast, in patients with a bifurcation angle of less than 80°, irregular VIE appearances were only noticed in 1 patient (1/19).

## 4. Discussion

This study provides an insight into the intraluminal appearances of coronary plaques and corresponding coronary wall changes in relation to the left coronary angulation and types of plaques. VIE, as a novel visualization tool, clearly demonstrates the intraluminal changes of different types of plaques and offers additional information compared to conventional 2D or 3D extraluminal views. Results of this study indicate that there is a direct and strong correlation between the VIE appearances of coronary lumen and different types of plaques, while the left coronary artery with wide angulation relates to the subsequent coronary wall changes as shown in the VIE visualization, although this is only observed in about 50% of the patients.

It is widely acknowledged that unstable (or vulnerable) coronary plaques play a pivotal role in the pathogenesis of acute coronary syndromes due to local thrombus formation caused by plaque rupture or erosion. Therefore, plaque composition rather than the degree of luminal narrowing may be predictive of the patient's risk for further coronary events [7]. Extensively calcified lesions most likely represent atherosclerosis at later stages of remodeling and may reflect more stable lesions [17]. However, earlier stages of atherosclerosis which lack the presence of calcium deposits may be more prone to rupture with subsequent occurrence of acute events. Consequently, it is clinically important to characterize the coronary plaques, especially identification of the unstable plaques, rather than detect the degree of coronary stenoses. This is confirmed in this study as plaque components are closely related to the corresponding VIE changes of the coronary lumen. We believe that VIE could be used as a complementary tool to conventional visualization for accurate assessment of the coronary plaques by demonstrating the corresponding luminal changes to the coronary wall, although further studies based on a large cohort are needed to support our preliminary findings.

In this study, characterization of the coronary plaques intraluminally was presented with demonstration of variable appearances, corresponding to the type and location of the plaques. Results of this study confirm that most of the calcified plaques show smooth protruding sign surrounding the coronary ostium, as the majority of calcified plaques are ostial lesions, located in the proximal regions of the coronary arteries, particularly in the LAD (Figures 7, 8, 11, 12, and 14). Our findings are consistent with other reports regarding the distribution and morphology of coronary plaques [4, 18, 19]. Atherosclerotic plaques were commonly located at the ostial or proximal LAD, whereas the left main stem and the ostial LCx were less frequently affected.

A bifurcation angle of 80° is recommended as a cut-off value to determine whether there is presence of coronary artery disease, as this was confirmed by previous studies investigating the natural distribution of coronary bifurcation angles [20, 21]. Reig and Petit reported an average angle of 86.7° ± 28.8° for the bifurcation angle between LAD and LCx based on autopsies of 100 human hearts [20]. Pflederer et al. found an average bifurcation angle of 80° ± 27° in 100 patients with suspected CAD [21], and Kawasaki et al. reported that the LAD and LCx bifurcation angle was 72° ± 22° in 209 patients [22]. The presence of high-risk and noncalcified plaques has been reported to be associated with higher bifurcation angles [23]. Measurements of bifurcation angle in this study are in line with the average bifurcation angle reported by these studies, with diseased LAD having left coronary angulation of more than 80° (mean angle: 94.3°), while the normal LAD having angulation of less than 80° (mean angle: 76.5°). This strong relationship between the angle of the left coronary artery and the presence of plaques within the left coronary bifurcation could be used as guidance for analysis of potential effects of the hemodynamics on the local characteristics and distribution of plaques [24–29], although further studies are warranted to evaluate the potential value of these findings.

Despite the strong relationship between left coronary bifurcation angle and dimensional changes, this study shows that irregular VIE appearances were only observed in about 50% of patients with angulation of more than 80°, while, in patients with mixed type of plaques, the irregular VIE appearances were noticed in more than 90% of patients. This emphasises the fact that plaque components rather than the degree of stenosis or bifurcation angle directly affect the coronary lumen changes. Recently, coronary CT angiography has been proposed as a promising noninvasive modality for identification of plaque patterns and characteristics and associated cardiac events [16, 30–34]. Motoyama et al. showed that plaques with low CT attenuation and positive remodelling were at particularly high risk for rupture [30]. Kitagawa et al. studied 228 noncalcified plaques in their cohort of consecutive 147 patients and correlated the prevalence of noncalcified plaques with acute coronary syndrome using 64-slice coronary CT angiography [31]. Their results showed that 64-slice coronary CT angiography allowed characterization of plaques with more noncalcified plaques associated with plaque vulnerability in patients with acute coronary syndrome when compared with patients presenting with stable clinical presentation. Thus, analysis of the plaque components plays an essential role in risk stratification of patients with coronary artery disease. We believe that the novel visualization tool, VIE, may be useful for noninvasive identification of plaque characteristics associated with high risk by demonstrating the patterns of intraluminal coronary lumen changes, although prospective studies in patients with different risks are needed to confirm these observations.

Several limitations in this study must be acknowledged. Firstly, coronary CT angiography was not compared with invasive coronary angiography for the diagnosis and measurement of CAD. This limitation can be compensated for by the fact that coronary CT angiography was confirmed as an accurate tool to measure coronary bifurcation angles [21]. The purpose of this study was to demonstrate the diagnostic value of VIE in the visualization of coronary plaques in terms of intraluminal appearances instead of diagnostic accuracy. Secondly, the relationship between calcified and noncalcified plaques with patients' symptoms or cardiac events was not studied. Further studies of correlating VIE findings of these plaques with subsequent major adverse cardiac events are needed. Finally, the sample size of this study is small, as only 33 patients were found to have coronary artery disease.

In conclusion, this study shows the potential applications of using 3D VIE visualization for assessment of coronary plaques in terms of characterization and intraluminal appearances of the artery wall. A direct correlation is found between the types of coronary plaques and corresponding VIE appearances of the coronary lumen. Despite a preliminary study without strong evidence of clinical correlation, the results are considered valuable for enhancing our understanding of the effect of different types of coronary plaques on coronary wall and subsequent clinical outcomes. VIE visualization could serve as a complimentary tool to conventional image visualization for the detailed analysis of coronary lumen changes in relation to the plaque components, therefore, assisting identification of unstable or vulnerable plaques.

## Figures and Tables

**Table 1:** Measurements of left bifurcation angle and left coronary diameters with corresponding VIE appearances.

Patients	Age/sex	Diameter of left coronary artery		Bifurcation angle (°)	Types of plaques			VIE appearances	
		LAD (mm)	LCx (mm)	LAD-LCx	Calcified	Noncalcified	Mixed	LAD	LCx
1	72/F	3.4	3.6	110.3	LAD/LCx	—	LCx	Irregular	Smooth
2	59/F	3.7	3.2	101	LAD/LCx	—	—	Smooth	Smooth
3	59/M	4.0	3.1	90.9	LAD/LCx	—	—	Irregular	Smooth
4	52/M	6.1	3.7	134.5	LAD/LCx	—	LAD	Irregular	Smooth
5	38/M	3.0	1.6	55.3	LAD	—	LAD	Irregular	Smooth
6	53/F	3.5	2.0	73	LAD	—	—	Smooth	Smooth
7	56/F	2.5	2.2	73.9	—	—	—	Smooth	Smooth
8	49/M	2.7	1.5	96.1	—	—	—	Smooth	Smooth
9	51/M	3.2	2.4	88.8	LAD	—	—	Smooth	Smooth
10	69/M	5.3	2.5	112.8	LAD	—	LAD	Irregular	Smooth
11	55/M	3.8	3.5	111.3	LAD/LCx	—	—	Smooth	Smooth
12	58/M	4.1	2.9	73.5	LAD	—	—	Smooth	Smooth
13	56/M	3.0	2.9	69.1	LAD/LCx	—	—	Smooth	Smooth
14	42/M	3.9	2.9	124	LAD	—	—	Smooth	Smooth
15	56/F	3.9	3.1	105.1	LAD/LCx	—	—	Irregular	Irregular
16	61/M	4.1	2.8	92	LAD	—	LAD	Irregular	Smooth
17	55/M	4.3	3.8	97	LAD/LCx	—	LAD	Irregular	Smooth
18	57/F	3.6	2.9	76.1	LAD	—	LAD	Smooth	Smooth
19	43/F	5.2	3.9	114.5	LAD/LCx	—	LAD	Irregular	Irregular
20	45/M	2.8	2.6	104.4	LAD	—	—	Smooth	Smooth
21	65/F	2.2	2.0	82	—	—	—	Smooth	Smooth
22	60/M	4.4	2.9	75.3	LAD	—	—	Smooth	Smooth
23	61/M	4.5	3.2	77	LAD	—	—	Smooth	Smooth
24	66/F	4.8	3.2	91	LAD	—	—	Irregular	Smooth
25	58/M	5.1	3.8	92	LAD	—	—	Irregular	Smooth
26	63/M	2.7	2.4	70	—	—	—	Smooth	Smooth
27	55/F	3.2	2.9	60	—	—	—	Smooth	Smooth
28	47/F	3.2	2.3	72	—	—	—	Smooth	Smooth
29	65/M	2.8	2.0	90	—	—	—	Smooth	Smooth
30	65/F	3.1	1.9	60	—	—	—	Smooth	Smooth
31	60/M	3.6	3.0	81	LAD/LCx	—	—	Smooth	Smooth
32	53/M	4.7	3.4	99.3	LAD	—	—	Smooth	Smooth
33	52/M	3.2	2.6	77.3	—	—	—	Smooth	Smooth
34	64/F	4.2	2.7	101	—	—	—	Smooth	Smooth
35	58/F	4.6	2.4	88.5	LAD/LCx	—	—	Irregular	Irregular
36	46/F	3.6	2.8	72	—	—	—	Smooth	Smooth
37	49/M	3.4	2.4	83.5	—	—	—	Smooth	Smooth
38	53/M	5.7	2.5	97.7	—	—	LAD/LCx	Irregular	Irregular
39	54/F	4.0	2.9	40.3	—	—	—	Smooth	Smooth
40	51/F	4.1	3.4	78.7	LAD	—	—	Smooth	Smooth
41	47/M	4.8	4.0	94.4	—	—	LAD/LCx	Irregular	Irregular
42	37/M	3.2	2.7	77.4	—	—	—	Smooth	Smooth
43	46/M	3.4	3.1	71.3	—	—	—	Smooth	Smooth
44	73/M	5.4	4.2	86.5	—	—	LAD/LCx	Irregular	Irregular
45	48/M	4.5	4.1	102.2	LAD	—	—	Smooth	Smooth
46	46/M	4.0	2.9	105	—	—	—	Smooth	Smooth
47	46/F	3.3	2.8	70.3	—	—	—	Smooth	Smooth
48	71/M	3.7	3.4	104.5	LAD	—	—	Smooth	Smooth
49	74/F	3.9	2.3	96.8	—	LAD	—	Smooth	Smooth
50	63/M	4.2	3.7	105.6	LAD	—	—	Irregular	Smooth

LAD: left anterior descending; LCx: left circumflex; VIE: virtual intravascular endoscopy.
